# Erector spinae plane block versus paravertebral block in breast surgeries

**DOI:** 10.1186/s12871-022-01946-5

**Published:** 2022-12-28

**Authors:** Raghuraman M. Sethuraman

**Affiliations:** Department of Anesthesiology, Sree Balaji Medical College & Hospital, BIHER, #7, Works Road, New Colony, Chromepet, Chennai, 600044 India

**Keywords:** Erector spinae plane block, Paravertebral block, Modified radical mastectomy

## Abstract

This article (Correspondence) is in response to the recently published study by Elewa et al. in BMC Anesthesiology that compared the erector spinae plane block (ESPB) versus paravertebral block (PVB) regarding postoperative analgesic consumption following breast surgeries. I greatly appreciate the authors for publishing this study which is one among a very few studies available on this topic. I wish to present my reflections on this article as well as add a few more points on this topic.

**Dear Editor,**

I read with great interest the recently published study that compared the erector spinae plane block (ESPB) vs paravertebral block (PVB) for modified radical mastectomy (MRM) procedures [1]. I congratulate Elewa et al. [1] for this wonderful study that is one among the very few studies comparing these 2 regional techniques in breast surgeries and wish to add a few more points.

Elewa et al. [1] concluded that ESPB and PVB were equally effective in reducing morphine consumption and stated in the discussion section that this “potentially stems from its ease of performance with no major technical difficulties compared with the PVB and the widespread cutaneous sensory block by the ESPB may represent another mechanistic explanation of the present findings”. However, I believe that both these techniques provide almost similar sensory coverage for breast surgeries as observed in the current study by Elewa et al. [1]. PVB does not cover supraclavicular nerves, pectoral nerves, or other brachial plexus nerves [2]. ESPB, which is considered a technical modification of PVB (“Backdoor” entry to PVB [3]), also does not cover these nerves if performed at the mid-thoracic level as is commonly practiced for breast surgeries. The exact mechanism of action of ESPB is still “elusive” [3] because of its complexities that involve multidirectional spread of the injectate [4]. The main advantage of ESPB is that it is easier to perform and safer when compared to PVB, hence; does not require much expertise. However, Elewa et al. [1] incorrectly stated that “ESPB can be utilized in low-resourced facilities” as the resources (ultrasound machine, probes, needles) required are the same for both and only the level of expertise required is lesser for ESPB.

Although a few previous studies concluded that both ESPB and PVB were equally effective as mentioned by Elewa et al. [1], Swisher et al. [5] observed that PVB was superior to ESPB in non-mastectomy breast surgeries. Swisher et al. [5] stated that the reasons for the different conclusions between their study and the previous study by Gürkan et al. [6] (published in 2020 not in 2017 as stated by Elewa et al. [1]) were mainly the type of surgeries, block technique, volume and concentration of local anesthetic used. Swisher et al. [3] specifically stated the PVB technique adopted by Gürkan et al. [6] appeared to be “more lateral” rather than just lateral to the lamina at the level of the transverse process. Another study (retrospective) by Aoyama et al. [7] also observed that ESPB was associated with lesser dermatomal coverage besides lack of consistent sensory blockade when compared to PVB in breast surgeries.

Elewa et al. [1] mentioned in the discussion section that there were no significant differences between the ESPB and PVB for postoperative analgesia in breast surgeries as per a recent systematic review and meta-analysis and cited reference # 26 for that. However, that referenced study by Schnabel et al. [8] included only PVB and was published in 2010 hence, no possibility of comparing it with ESPB, as this technique was described only in 2016. Elewa et al. [1] could have cited the meta-analysis by Weng et al. [9], published in 2021, for that statement.

## Data Availability

Not applicable.

## Abbreviations

ESPB: Erector spinae plane block
PVB: Paravertebral block
MRM: Modified radical mastectomy
